# Nurses’ knowledge and their role in selected hospital logistics processes: a cross-sectional study

**DOI:** 10.1186/s12912-025-02812-8

**Published:** 2025-02-14

**Authors:** Katarzyna Jarosz, Elżbieta Czech, Joanna Jaromin

**Affiliations:** 1 Department of Gerontology and Geriatric Nursing, Faculty of Health Sciences, Medical University of Silesia, Katowice, ul. Ziołowa 45/47, Katowice, 40-635 Poland; 2 Department of Nursing and Social Medical Problems, Faculty of Health Sciences, Medical University of Silesia, Katowice, Poland; 3 Department of Biostatistics, Faculty of Health Sciences in Bytom, Medical University of Silesia, Katowice, Poland

**Keywords:** Logistics, Logistics processes, Nurses’ knowledge, Nurses’ role, 7Rs principle

## Abstract

**Introduction:**

Nursing as a profession is still changing, developing and seeking a place in the healthcare system. It also defines a new tasks and competencies in response to increasing patients’ needs and changing environment. Nursing tasks include not only strictly clinical tasks, but also non-nursing tasks that are necessary to carry out the treatment process such as logistics processes. The main aim of the study was to assess the level of knowledge of nurses about logistics processes and to assess the perception of the role of a nurse in selected hospital logistics processes. An additional objective was to assess the frequency of application of the 7R principle (the right product, in the right quantity, in the right condition, to the right place, at the right time, for the right customer/patient, at the right cost).

**Materials and methods:**

This is a cross-sectional study. In the study, 152 nurses participated. The original questionnaire included a sociodemographic and logistical section, which consisted of questions about the definition of the logistics process and its understanding, respondents’ participation in logistics processes, and the occurrence of logistics processes in the workplace. The nurses’ knowledge was assessed through five questions, and the results could range from 1 to 6 points. Four questions were used to analyze the role of nurses in logistics processes, with possible scores ranging from 2 to 9 points. The frequency of the 7Rs principle usage ranged from 0 to 35 points.

**Results:**

The level of respondents’ knowledge was moderate, with an average score of 4.67 points, and more than half of the nurses were characterized by a high level of knowledge about logistics processes (51.32%). The role of the nurse in the logistics process was assessed as moderately important, with a score of 5.91 points. The 7Rs principle was applied ‘sometimes,’ achieving a score of 26.67 points.

**Conclusions:**

Although logistics as a science is not widely included in nursing education and practice, respondents reported a moderate level of knowledge and assessed the role of nurses in logistics processes as moderately significant, suggesting that logistics is important in the work of nurses.

**Supplementary Information:**

The online version contains supplementary material available at 10.1186/s12912-025-02812-8.

## Introduction

Nurses’ tasks are primarily related to the implementation of care, nursing, treatment, diagnostics, rehabilitation, rescue activities, etc., as part of an independently performed profession or the execution of doctors’ orders. Nurses can also hold management and leadership positions [1]. The profession of a nurse requires professional education and skills; however, the work of a nurse also includes activities that do not require professional education in nursing. Grosso et al. in their research indicated that 94.5% of nurses reported performing non-nursing tasks, with 72.4% related to administrative matters, 66.7% involving auxiliary tasks, and the remainder associated with tasks undertaken by professionals from other medical fields [2]. Examples of these tasks include arranging discharges and transportation, managing equipment and supplies, answering phone calls, and handling office matters [3]. Additionally, they involve reporting equipment faults, preparing monthly orders and supplies for the entire hospital ordering system, overseeing stock, carrying samples to the laboratory, preparing disposable equipment, replenishing stock, and preparing documents and making copies [4].

A wide range of logistics activities and processes in hospitals has been shown in the literature, but they are not the hospital’s main mission; however, they are necessary to provide patient care and determine service levels [5, 6]. Logistics includes all activities related to the handling of flows—material, people, and information—that accompany the treatment process. In a narrow sense, hospital logistics includes only actions related to material supplies that enable the implementation of basic medical activities. However, in a broader sense, it encompasses activities related to services for patients, such as transportation, or indirect activities like the supply of medicines, auxiliary actions such as providing bedding, and activities that are invisible to patients, such as managing patients’ stays in the hospital [7]. There are not only medical processes and those directly connected to them but also management processes, such as material and information processes. These processes are strictly related to planning, organizing, and creating flows of materials, people, or information, which lie at the essence of logistics [8, 9]. The goal of logistics processes is to place the right items in the right location at the right time for the right medical staff or patient while maintaining the right (expected) condition and quantity, according to the 7Rs principle [8, 10]. Therefore, the goal of hospital logistics can be defined as the coordination of processes that support the main process, which is the treatment process, and the implementation of patients’ rights [11].

Canadian researchers Belanger et al. noted that managing supplies and their locations on wards posed challenges for hospital functioning, resulting in nurses spending a significant portion of their time on these tasks instead of providing direct patient services [12]. A large part of nurses’ responsibilities includes managing human and material resources, considering the individual needs of patients, assessing appropriate and optimal locations, and effectively using the IT system [13]. Michel et al. showed that nurses spend a significant portion of their time at work on various tasks that are not considered care activities, such as logistics and stewardship tasks, transporting or transferring patients, and then administrative tasks. Care planning was facilitated by the IT system, patient interviews, nursing handovers, communication and coordination activities, and team conferences were also an appreciable part of nurses’ work [14]. As the researchers showed, nurses spend a large amount of their time managing material and human resources, information flows, transporting, coordinating actions, and broadly understood administrative tasks. However, no research has described what kinds of logistics processes nurses perform, how they perceive their role, how much knowledge they have, and whether they are aware that logistics processes exist in their workplace. Researchers did not study why nurses spend so much time on these tasks, whether the reason is a lack of knowledge, inappropriate preparation of processes, or insufficient separation of duties.

Observations from work on the hospital ward and conversations with nurses reveal that the term “logistics” is abstract to them; they do not apply it to their work, even though they often participate in it—either consciously or subconsciously—and can influence the creation of these processes. Additionally, on average, nurses perform 72.3 tasks per hour, perform many tasks at the same time for 34% of their work time, and they are disturbed by coworkers or patients every 6 min [15]. That’s why nurses have difficulties with engagement in deep reflection and critical analysis [16], which causes them not to notice either the logistics processes or their role and impact on them. Moreover, logistics processes are neither planned nor supervised.

As Volland et al. showed, logistics functions have gained a strategic place in hospital management over the last 15 years [17]. This has helped to reduce errors, improve process quality, and shorten waiting times. Due to the complexity of hospital systems, as well as the variability and unpredictability of patient cases, logistics is considered an effective solution in organizing the work time of nurses, which provides them the opportunity to focus on their professional tasks related to patient care and improve the quality of care [18]. This demonstrates that it is indisputable that logistics processes exist in hospitals and within the work of nurses. This is why the authors decided to conduct research that assesses nurses’ knowledge of logistics processes, how they perceive their role in these processes, and whether they are able to apply this knowledge in their work after learning the definition. Therefore, the main aim of the study was to assess the level of nurses’ knowledge about logistics processes and their role within the hospital. The specific aims were to assess the frequency with which nurses use the 7Rs principle in their work, the correlation between the level of nurses’ knowledge, their role, the frequency of using the 7Rs principle, education, continuing professional development, understanding of the logistics process definition, workplace participation, and involvement in the logistics process before and after reading the logistics definition.

## Methods

### Design

The studied group includes 152 nurses from all over Poland working in various positions across different types of medical facilities. The study was conducted during the COVID-19 pandemic, and the research group consisted of all nurses who wanted to complete the survey at that time, making it very difficult to recruit respondents. The consent of the bioethics committee was obtained (Ethical Number: BNW/NWN/0052/KB/216/24). The study protocol was created in accordance with the Declaration of Helsinki.

### Inclusion and exclusion criteria

The inclusion criteria were being a nurse and providing voluntary consent to participate in the study, regardless of place, time, or type of employment. The exclusion criteria were a lack of consent to participate and incomplete responses to the questionnaire.

### Data collection and statistical procedures

Data were collected over three months, from October to December 2021, by sharing an online survey. All participants in the survey provided informed consent to complete it, and they were informed that participation in the study was voluntary and anonymous. Data were obtained from 152 nurses, but 141 respondents (92.76%) completed the form entirely. Participants could complete the survey early if, after reading the definition of the logistics process, they still did not understand it. Data from completed questionnaires were collected in an Excel spreadsheet. Statistical analyses were performed using the Statistica (version 13.3) program. Basic descriptive statistics, the Shapiro-Wilk distribution normality test, a series of correlation analyses with the Pearson r coefficient, the Tau Kendall test, the Kruskal-Wallis test, and a variance test were performed using this program. The Fisher test and Chi-square tests (Pearson and with Yates correction) were used to analyze the correlation between quantitative and qualitative data. The level of significance in this chapter was considered to be α = 0.05. Cronbach’s Alpha in the present study was 0.75 for the frequency of the 7Rs principle usage, 0.76 for assessing the nurse’s role in logistic processes, and 0.74 for the level of nurses’ knowledge.

### Instrument

The original, anonymous questionnaire was used to conduct the study. The questionnaire was administered in Polish. This survey was created specifically for this study, and it has not been published elsewhere; it was included as a supplementary file. It was divided into a sociodemographic part and a logistics part, which was further divided into two sections. The first section included questions regarding the definition of the logistics process and whether the respondent participates in logistics processes. The definition of the logistics process was then presented. The second section focused on understanding the definition of the logistics process and what it entails (if the respondent did not know it from the earlier question) and included questions about the occurrence of logistics processes in the workplace.

The nurses’ knowledge was assessed through five questions from the survey: What definition do you think describes the logistics process? Do you know what the logistics process is? Is the logistics process something that occurs in a hospital? Are you involved in the hospital’s logistics processes? Do you think logistics processes should be planned? For the first question, respondents could receive between 0 and 2 points, while for the remaining questions, they could receive between 0 and 1 point. The knowledge level was calculated by summing the points from these questions. The maximum score was 6 points, and the minimum was 0 points. Respondents who received a score of 0 points were excluded from further analysis, as they demonstrated no knowledge of the subject. The level of knowledge was determined according to the following scale: 1–2 points = low knowledge level; 3–4 points = moderate knowledge level; 5–6 points = high knowledge level.

Four questions were used to analyze the role of nurses in logistics processes: Question 18 - In your opinion, what part of the activities you perform constitutes logistics processes? 0 points– does not constitute; 1 point– a very small part; 2 points– a small part; 3 points– a moderate part; 4 points– a large part; 5 points– a very large part. Question 19– Who do you think performs the most activities related to logistics processes? 1 point– a nurse/a head nurse; 0 points– other answers. Question 21– In your opinion, how important is the role of a nurse in logistics processes? 0 points– not important; 1 point– very unimportant; 2 points– not very important; 3 points– important; 4 points– very important. Question 23– Who is responsible for planning logistics processes? 1 point– a head nurse/a coordinating nurse/a person directly involved in the logistics process; 0 points– others. The level of the nurse’s role in logistics processes was calculated by summing the points from these questions. The maximum score was 9 points, while the minimum score was 0 points. Respondents who had previously completed the survey were excluded from the analysis. The level of the nurse’s role was determined according to the following scale: 2–3 points– not very important; 4–5 points– moderately important; 6–7 points– important; 8–9 points– very important.

The frequency of the 7Rs principle’s usage was also calculated by summing points. Participants could score between 0 and 5 points for each answer, resulting in a total score range of 0 to 35 points. The analysis excluded 11 respondents who had previously completed the survey. The frequency of the 7Rs principle’s usage was determined according to the following scale: 0–24 points– rarely or very rarely; 25–28 points– sometimes; 29–30 points– often; 31–35 points– very often or always.

## Results

### Characteristics of the sample

In the study, 152 nurses participated, including 150 women (98.68%) and two men (1.32%). The mean age was 36.95 years (± 10.83 years), and the mean seniority in the nursing profession was 12.74 years (± 11.49 years). In the city, 118 respondents (77.63%) lived, while 34 respondents (22.37%) resided in the village. The majority of the studied group consisted of married nurses (62.50%; 95 respondents). Most nurses worked in a 12-hour shift system (125 respondents; 82.24%). A bachelor’s degree was held by 111 nurses (73.03%), a master’s degree by 37 nurses (24.34%), and only 4 nurses (2.63%) had a secondary education. One form of continuing professional development was completed by 47 nurses (30.92%), two forms by 33 nurses (21.71%), three forms by 24 nurses (15.79%), and 39 nurses had no additional forms of continuing professional development. However, 9 nurses (5.92%) had four or more additional forms of continuing professional development. The largest group of respondents worked in hospital wards—128 nurses (84.21%); among them, there were primarily non-surgical departments (81 nurses; 53.29%) and surgical departments (43 nurses; 28.29%).

### Normality test

The Shapiro-Wilk test results were statistically significant for each variable. This means that the distributions of these variables deviate statistically from the normal curve. Therefore, non-parametric tests were used for further analysis [23]. Details are presented in Table 1.

**Table 1:** The Shapiro-Wilk distribution normality test

Variable	Number of respondents	Shapiro-Wilk test	Significancy level
Seniority	141	W = 0.85	*p* < 0.00001
Knowledge level	141	W = 0.88	*p* < 0.00001
Level of nurse’s role	141	W = 0.92	*p* < 0.00001
7R principle	141	W = 0.85	*p* < 0.00001

Source: Own study

### Descriptive analysis

The respondents’ knowledge level was moderate, scoring 4.67 points (± 0.97 points). The role of the nurse in the logistics process was rated as moderately important with a score of 5.91 points (± 1.51 points), and the 7Rs principle was applied ‘sometimes’ with a score of 26.67 points (± 7.13 points). Details are presented in Table 2.

More than half of the participants (51.32%; 78 respondents) had a high level of knowledge about hospital logistics processes. Only 6 respondents (3.95%) had a low level of knowledge, while 8 respondents (5.30%) had no knowledge, scoring 0 points. The majority of respondents—71 nurses (50.35%)—believed that the nurses’ roles in the logistics process were important; 17 (12.06%) nurses believed that they were very important, and only 7 (4.96%) nurses believed that they were of little importance. The largest group of respondents—40 nurses (28.37%)—used the 7Rs principle very often or always, while the smallest group—27 nurses (19.15%)—used it sometimes. Details are presented in Table 2.

**Table 2:** Characteristics of the studied group including the knowledge level, the nurse’s role in the logistics process and the usage of the 7R principle

Variable	*N*	M	SD	Me	D	Min.	Max.	D^2^
Knowledge level	141	4.67	0.97	5.00	4.00	2.00	6.00	0.94
Nurse’s role	141	5.91	1.51	6.00	6.00	2.00	9.00	2.29
7R principle	141	26.67	7.13	29.00	30.00	3.00	35.00	50.91
Seniority	141	12.70	11.45	8.00	1.00	0.60	36.00	131.06
**The knowledge level**							**N**	**%**
Low							6	3.95
Moderate							60	39.47
High							78	51.32
Excluded from analysis (0 points)							8	5.26
**Role of the nurse**								
Not very important							7	4.96
Moderately important							46	32.62
Important							71	50.35
Very important							17	12.06
**Frequency of the 7R usage**								
Very often or always							40	28.37%
Often							38	26.95%
Sometimes							27	19.15%
Rarely or very rarely							36	25.53%

Abbreviation: N– number of respondents; M– average; Mdn– median; SD– standard deviation; Min. and Max. - the smallest and largest value of the distribution; D^2–^ variance

Source: Own study

### Correlation analysis

The results of the Spearman correlation analysis (*p* = 0.0007) showed that the perception of the nurse’s role in the logistics process has a statistically significant impact on the 7Rs principle. There is a weak positive relationship between the perception of the nurse’s role and the application of the 7Rs principle. This indicates that a better perception of the nurse’s role in the logistics process correlates with more frequent use of the 7Rs principle in practice. Details are presented in Table 3.

The Chi-squared test with Yates’ correction showed a weak relationship between knowledge of the definition of the logistics process before and after reading it. Fisher’s exact test indicated a weak relationship between the use of the 7Rs and the workplace. The remaining tests did not show any statistically significant relationships. Details are presented in Table 3.

**Table 3:** Correlation between the usage of the 7R principle, the knowledge level and the nurse’s role in the logistics process

Dependent variable	Independent variable	Kruskal-Wallis test	*p*	Variance test	*p*	tau Kendall coefficient	*p*
7R principle	Knowledge level	H = 1.34	*p* = 0.51	χ^2^ = 0.41	*P* = 0.81	τ = 0.08	*p* = 0.16
	Nurse’s role	H = 10.16	*p* = 0.02	χ^2^ = 10.05	*P* = 0.02	τ = 0.18	*p* = 0.0012

Abbreviation: p– significance

Source: Own study

In the participants’ opinion, the person who performs the majority of activities connected with logistics processes is a head nurse (88 respondents; 62.41%) and a nurse (39 respondents; 27.66%). More than half of the respondents declared that, in their workplace, part of the logistics processes is planned (80 respondents; 56.74%), and they indicated the head nurse as the person responsible for planning logistics processes (77 respondents; 54.61%). Details are presented in Table 4

**Table 4:** Correlation between the knowledge of the logistics process definition, the participation in logistics processes, the application the 7R principle, the knowledge level, the nurse’s role in the logistics process, workplace, education

Pair of variables		Test	Dependence	*p*
Knowledge level	Seniority	R_S_= 012	t(N-2) = 1.27	0.21
	Role of the nurse	R_S_= 016	t(N-2) = 1.97	0.05
	7R principle	R_S_= 0.12	t(N-2) = 1.39	0.17
Role of the nurse	Seniority	R_S_= 0.02	t(N-2) = 0.23	0.82
	7R princicple	R_S_= 0.28	t(N-2) = 3.45	0.0007
7R principle	Seniority	R_S_= -0.07	t(N-2) = -0.85	0.4
Participation in the logistics process before reading the definition	Participation in the logistics process after reading the definition	Fisher = 4.52	V Cramer = 0.2	0.10
Knowledge of the definition before reading it	Knowledge of the definition after reading it	Chi^2^ (with Yates correction) = 12.6	V Cramer = 0.28	0.002
Application of the 7R principle	Knowledge of the definition before reading it	Chi^2^ (Pearson) = 1.15	V Cramer = 0.09	0.77
	Workplace	Fisher = 29.34	V Cramer = 0.25	0.0006
Knowledge level	Education	Fisher = 4.62	Tau c Kendall = -0.03	0.1
	Continuing professional development	Fisher = 6.74	Tau c Kendall = 0.16	0.35
Role of the nurse	Education	Fisher = 3.28	Tau c Kendall = 0.14	0.35
	Continuing professional development	Fisher = 10.26	Tau c Kendall = 0.05	0.33

Abbreviation: Rs– R Spearman tes; p - significance

Source: own study

## Discussion

Respondents assessed the nurse’s role in the logistics processes as moderately important. Half of them believe that nurses’ roles are important, and they identified the head nurse as the person performing the most logistics activities and responsible for planning logistics processes. This demonstrates how crucial the nurse’s role is in logistics processes. Unfortunately, only a portion of the logistics processes was planned in the respondents’ workplaces. Most of them had a high level of knowledge about the logistics process, and they also frequently or always applied the 7Rs principle. This reflects the wide range of applications for logistics in nurses’ work and the importance of understanding logistics processes. The topic related to logistics and its dimensions in a nurse’s work has not been explored thus far. There have been many publications and dissertations on hospital logistics and the logistics processes occurring in hospitals, but none of them discuss the work of nurses or their participation in logistics processes.

A positive, statistically significant correlation was found between the frequency of applying the 7Rs principle at work and nurses’ perceptions of their roles in logistics processes. This may be due to the fact that if nurses perceive their roles in the logistics process more positively, they will be more motivated to apply the 7Rs principle at work and will experience a greater sense of agency. Additionally, there is a relationship between the frequency of applying the 7Rs principle and the workplace environment, as well as a relationship between knowledge of the definition of the logistics process before and after reading about it. These relationships may be influenced by various factors in the work environment, necessitating separate and extensive research. Nurses and midwives exhibit diverse attitudes toward their involvement in hospital activities. Younger professionals tend to be more engaged; they seek to apply their acquired knowledge and skills, strive to showcase their abilities, and continuously pursue personal development to attain improved living standards and achieve their professional objectives [19].

Nurses’ perceptions of their role in the logistics process have not been assessed so far, but the general perception of their profession has been evaluated. Dziubak and Motyka’s research, conducted among undergraduate nursing students, showed that the perception of the nursing profession has become increasingly negative over subsequent years of study [20]. Additionally, the study by Koralewicz et al. indicated that students consider the status of the nursing profession to be moderate (32.3%) and almost low (31.7%) compared to other medical professions [21]. These findings confirm those of Sykut et al., in which only 10% of nurses regard the professional status of a nurse as high or very high [22]. In our study, nurses rated their role in the logistics process as important (50.35%) and moderately important (32.62%). This assessment may stem from the general perception of one’s profession or a lack of ability to integrate logistics into nursing work. Moreover, the nursing profession is still burdened with many stereotypes and is often seen as subordinate to doctors, which hinders nurses from exercising their professional independence. Healthcare systems mostly recognize the concept of medical care, but nurses’ autonomy is frequently overlooked. This has resulted in nurses focusing solely on patient care and medical tasks, neglecting additional areas such as logistics that might enhance care [14].

According to the regulations of the Polish Health Minister dated October 29, 2003, each nurse completing a specialization in a selected field had to complete the so-called general module, which included, among other things, modules related to management and organization, including elements of health care economics [23]. However, according to the announcement of the Minister of Science and Higher Education dated January 9, 2018, every nurse completing a master’s degree must take classes in the subject “Management in Nursing” during their education [24]. The information above shows that nurses completing a specialization or a master’s degree, or both forms of education, should have a basic knowledge of management; this knowledge is not strictly related to logistics but can serve as a foundation for its introduction. Additionally, every Polish nurse is obligated to pursue professional development through courses, specializations, attendance at conferences, and postgraduate education, among other options. The studied group of nurses had knowledge of the logistics process at an average level of 39.47% and a high level of 51.32%. This could result from the education obtained by the nurses, although it was not demonstrated by the correlation tests in our own research.

Currently, bedside nurses work in environments where clinical knowledge and skills are insufficient to provide high-quality care due to barriers within complex and dysfunctional healthcare systems. That’s why nursing leadership must play a central role in improving quality outcomes to ensure the provision of high-quality bedside care [25]. Szołtysek and Twaróg argue that there is a deep-rooted belief that a healthcare manager should be someone from a medical profession, such as a nurse. Although these professionals possess highly specialized skills and knowledge in the field of medicine, they often lack expertise in management or logistics [26]. In contrast, West et al. and Rizany et al. describe a significant difference between nurse leaders who have completed management academic courses and those who have not. It is pointed out that management training for nurses should extend beyond the scope of nursing and include, for example, artificial intelligence, technology, logistics, and more [27]. This demonstrates that medical professionals may serve as managers, but they must be properly prepared, not only in the medical field but also in management.

In the current study, the majority of respondents identified the head nurse as the person responsible for planning logistics processes, followed by individuals directly involved in the logistics process. Those directly engaged in the logistics process may include any hospital employee, such as a nurse, doctor, cleaning staff, or secretary; however, not all of them may be responsible for planning the logistics process. An important consideration is the knowledge and skills of the person planning the logistics process, particularly in logistics and management, as well as their practical preparation. As mentioned above, a head nurse becomes a manager and takes responsibility for the proper execution of processes. Additionally, the selection of nurses may be influenced by perceptions of the head nurse as their immediate supervisor. Considering the tasks that nurse managers must perform, it can be concluded that many of their responsibilities relate to logistics processes and underscore the significance of the nurse manager in those processes. In her study, Majchrowska described the responsibilities of head nurses, which include, among other things, managing the daily replenishment of medicines and materials, coordinating repairs of medical and non-medical equipment, and verifying the accuracy of nursing tasks and medical orders performed by nurses, particularly regarding their punctuality and correct execution [28]. All of these tasks relate to logistics processes, and all nurses working in a department contribute to them, for example, by reporting damaged equipment to the head nurse, as confirmed by respondents. According to them, the head nurse (62.41%) was the person performing the most activities in logistics processes, while the nurse (27.66%) was the second most active individual in these processes. This emphasizes that the role of nurses in logistics is valuable and that at least basic knowledge in logistics is required of nurses, with advanced knowledge expected from their superiors to facilitate the effective operation of the department.

Planning is an inseparable part of the definition of logistics, and logistics cannot exist without planning. Unfortunately, processes within the company exist and take place even if they have not been identified and described. Many processes remain unnoticeable. Each process should be properly prepared in terms of both conceptual and substantive aspects [29]. Most nurses indicated that only some of the logistics processes in their workplaces are planned (56.74%), while slightly fewer nurses indicated that all logistics processes are planned (33.33%). The ideal situation would be for each process to be well thought out, planned, and optimized. It can be assumed that partial process planning may be influenced by the insufficient knowledge and competence of process managers or that the planning of logistics processes is not noticeable to respondents. More than half of the respondents (54.61%) indicated that the head nurse is responsible for planning the logistics process, which emphasizes the importance of their role in logistics processes and shows that, in healthcare facilities, nursing leaders are a crucial part of the therapeutic team. Nursing leaders are responsible for introducing changes and creating workplaces where nurses can provide high-quality care while simultaneously guaranteeing the facility’s aims. There is increasing awareness that the malfunction of quality and safety in healthcare is often caused more by systems and processes rather than by human errors. To address this, healthcare leaders are more frequently using management practices from other industries, such as Lean and Six Sigma, which are part of logistics [25].

Gawrońska stressed that the main task of logistics is to coordinate processes that support the therapeutic process and to meet the requirements of the five “rights” directly related to patients (the right patient, the right dose, the right medicine, the right time, and the right route of administration). This is often expanded by three additional components: the right documentation, the right basis for administering a medicine, and the right response from the patient’s body to the prescribed medicine [11]. It can be noted that the mentioned “authorities” overlap to some extent with the 7Rs principle and constitute the basis for performing nursing duties. In our study, the largest group of nurses (26.3%) used the 7Rs principle very often or always; however, an equal number of nurses used it very rarely or rarely (23.7%), which is a disturbing phenomenon and may be associated with insufficient rationalization of care. This situation also leads to medical errors that should not occur. Applying the 7Rs principle is extremely important to ensure patient safety and the proper performance of duties. Moreover, an effective logistics approach is based on the qualifications and skills of major participants (clients, leaders, nurses, etc.). The hospital should strengthen this aspect by creating an informative campaign and recruitment policies adapted to logistics requirements [12], which may increase the frequency of usage of the 7Rs principle.

Kautsch reckons that one of the main problems in Polish hospitals in the logistics area is the lack of standardization of logistics processes [30]. This indicates that the roles of nurses and their tasks in logistics processes are not clearly described. This situation requires extensive research and the preparation of detailed guidelines that can be included, for example, in the Act on the Professions of Nurses and Midwives. Research such as ours can contribute to the development and standardization of logistics processes. The management of logistics actions goes beyond traditional physical flows and includes other flows, such as patients within the entire care chain. Patient management entails several multidisciplinary and interdependent medical and administrative steps, which require controlled synchronization and interconnection to avoid issues, such as long waiting times or inappropriate usage of medical resources [12]. In the context of global healthcare transformation, nurses face increasing pressure to enhance the quality of care. They should be central to healthcare delivery to help medical facilities adapt to changes effectively. As highlighted, when nurses are involved in healthcare processes, including management and nursing care, organizations achieve better outcomes [27].

## Limitations of the study

The main limitation of the study is the lack of a standardized questionnaire; therefore, the authors created their own questionnaire. The limited number of studies on logistics in nursing represents a significant limitation, making comparisons between studies difficult and incomplete. The sample size was as large as we could recruit at that time (during the COVID-19 pandemic) and should be expanded in future studies. Despite these limitations, this study serves as a valuable introduction, highlighting the importance of logistical processes in nursing and demonstrating the need for more research in this field, along with the development of a standardized questionnaire.

## Conclusions

Respondents reported a moderate level of knowledge and assessed the role of nurses in logistics processes as moderately significant. The 7R principle was applied occasionally, indicating that logistics as a science should be integrated into nursing education and, subsequently, into practice, as there are considerable gaps in their knowledge and actions. A better understanding of logistics processes and the application of the 7Rs principle by nurses would enhance their work efficiency and save time that could be dedicated to patients.

Before taking any action, further studies are required to expand knowledge about the gaps in understanding and the perception of nurses’ roles in logistics processes. Logistics in nursing is inadequately examined, and it is essential to create a reliable, standardized, and international measurement tool. This instrument would help compare these elements among nurses in different countries and improve both the logistics process and the efficiency of nurses’ work.

In logistics processes, and more generally, in nursing logistics, there is a lack of overall standardization regarding who should be responsible for logistics processes, their development, and corrective actions. All processes should be carefully prepared, described, and executed according to instructions; only in this case would results be observable.

## Electronic supplementary material

Below is the link to the electronic supplementary material.

Supplementary Material 1

## Data Availability

The datasets used and/or analysed during the current study are available from the corresponding author on reasonable request.
