# Supplementary material for: Nurses’ knowledge and their role in selected hospital logistics processes: a cross-sectional study
Source: BMC Nurs. 2025 Feb 14;24:172. doi: 10.1186/s12912-025-02812-8 (PMC11827434; doi:10.1186/s12912-025-02812-8)
Supplement: Supplementary file 1 — Supplementary Material 1 [file 12912_2025_2812_MOESM1_ESM.docx]

Questionnaire

#### Metrics

1. Age: ……………..

| 2. Sex: | |
| --- | --- |
| □ | Female |
| □ | Male |

| 3. Place of residence: | |
| --- | --- |
| □ | City |
| □ | Village |

| 4. Marital status: | |
| --- | --- |
| □ | single |
| □ | Widow/ widower |
| □ | Non-formal relationship |
| □ | divorcee |
| □ | married |

5. Total length of service:

…................……………………………………………………………..

6. What department do you work in?…...........……………………………………………….

| 7. Position in which you work: | |
| --- | --- |
| □ | Nurse in hospital (what department?.......................) |
| □ | Management position |
| □ | Primary care/long-term care/specialist clinic nurse |
| □ | Nurse in nursing home, hospice, etc. |
| □ | Other (what?.......................…………………………………………………………....) |

| 8. Education: | |
| --- | --- |
| □ | medium |
| □ | Bachelor’s degree |
| □ | Master’s degree |
| □ | Doctoral degree |
| □ | Professor |

| 9. Postgraduate education: | |
| --- | --- |
| □ | Nursing Specialization (what?..........………………………………...……...) |
| □ | Nursing Qualification Course (what?…..........…………………………........................) |
| □ | Specialized course |
| □ | Postgraduate study (What? …..........………………………......................) |
| □ | Another field of study (What? …....................................…………………….) |
| □ | Not applicable. |

| 10. Operating mode: | |
| --- | --- |
| □ | 8-hour shift work |
| □ | 12h-hour shift work |
| □ | One-shift work |

#### Specialist part

| 11. Do you know what is the logistics process? | |
| --- | --- |
| □ | Yes |
| □ | No |

| 12. In your opinion, which definition could describe the logistics process? ? | |
| --- | --- |
| □ | The logistics process is an orderly and regulated sequence of activities closely related to the flow of materials, resulting in a specific effect. |
| □ | The logistics process is an orderly and regulated sequence of activities closely related to the flow of information, resulting in a specific effect. |
| □ | The logistics process is an orderly and regulated sequence of activities closely related to the flow of people, resulting in a specific effect. |
| □ | The logistics process is an orderly and regulated sequence of activities closely related to the flow of materials, resulting in an uncontrolled effect of action. |

| 13. While performing your duties at work, do you participate in logistics processes? | |
| --- | --- |
| □ | Yes |
| □ | No |
| □ | I don’t know, what Logistics process is |

**Definition:**

***The logistics process is an orderly and regulated sequence of activities closely related to the flow of materials, resulting in a specific effect.***

After reading the above definition of logistics process, please define:

| 14. Do you know what logistics processes are? | |
| --- | --- |
| □ | Yes, after reading the definition I now know what logistics processes are |
| □ | Yes, I knew it before |
| □ | No, I didn't know before and after reading the definition I still don't know what logistics processes are |

*(If you selected “no” in the above question, please stop completing the survey)*

| 15. In your opinion, is the logistics process something that occurs in a hospital? | |
| --- | --- |
| □ | Yes |
| □ | No |

| 16. Are you involved in the hospital's logistics processes? | |
| --- | --- |
| □ | Yes |
| □ | No |

| 17. In your opinion, should logistics processes be planned? | |
| --- | --- |
| □ | Yes |
| □ | No |
| □ | I don’t know |

| 18. In your opinion, what part of the activities you perform constitute logistics processes?  □ none  □ a very small part  □ a small part  □ a moderate part  □ a large part  □ a very large part |
| --- |

;

| 19. Who do you think performs the most activities related to logistics processes? | |
| --- | --- |
| □ | Doctor |
| □ | Nurse |
| □ | Medical caregiver |
| □ | Head nurse |
| □ | Cleaning person |

20. Please rate, on a scale of 0 to 5, how often you apply the 7R principle in your work and evaluate its individual elements. When assessing, please mainly consider the application of this principle to the use, transport, storage, etc., of materials in the ward.

| 7R principle | 0 - never | 1 – really rare | 2 - rare | 3 - sometimes | 4 - often | 5 - always |
| --- | --- | --- | --- | --- | --- | --- |
| Right product/drug |  |  |  |  |  |  |
| Right quantity/ dose |  |  |  |  |  |  |
| Right condition |  |  |  |  |  |  |
| Right place |  |  |  |  |  |  |
| Right time |  |  |  |  |  |  |
| Right patient |  |  |  |  |  |  |
| Right price |  |  |  |  |  |  |

| 21. In your opinion, how important is the role of a nurse in logistic processes? | |
| --- | --- |
| □ | not important |
| □ | very unimportant |
| □ | Not very important |
| □ | important |
| □ | very important |

| 22. Does your workplace plan logistics processes? | |
| --- | --- |
| □ | Yes, all of them are carefully planned |
| □ | Yes, only some of them are planned |
| □ | No, no logistics process is planned |

If the answer to the previous question was YES, please answer the following quest

| 23. Who is responsible for planning logistics processes? | |
| --- | --- |
| □ | Person directly involved in this process |
| □ | Head nurse |
| □ | Nurse coordinator |
| □ | Doctor |
| □ | Non-medical management |
